# The case for decoupling assembly and submission standards to maintain a more flexible registry of biological parts

**DOI:** 10.1186/1754-1611-8-28

**Published:** 2014-12-01

**Authors:** Razan N Alnahhas, Ben Slater, Yunle Huang, Catherine Mortensen, Jordan W Monk, Yousef Okasheh, Marco D Howard, Neil R Gottel, Michael J Hammerling, Jeffrey E Barrick

**Affiliations:** Department of Molecular Biosciences, Institute for Cellular and Molecular Biology, Center for Systems and Synthetic Biology, The University of Texas at Austin, Austin, Texas 78712 USA

**Keywords:** Synthetic biology, Biological part, DNA assembly, Gibson assembly, Gene synthesis, BioBrick, Registry of standard biological parts, Assembly standard, Submission standard

## Abstract

The Registry of Standard Biological Parts only accepts genetic parts compatible with the RFC 10 BioBrick format. This combined assembly and submission standard requires that four unique restriction enzyme sites must not occur in the DNA sequence encoding a part. We present evidence that this requirement places a nontrivial burden on iGEM teams developing large and novel parts. We further argue that the emergence of inexpensive DNA synthesis and versatile assembly methods reduces the utility of coupling submission and assembly standards and propose a submission standard that is compatible with current quality control strategies while nearly eliminating sequence constraints on submitted parts.

The Registry of Standard Biological Parts (hereafter, the Registry) aims to provide a collection of well-characterized genetic parts (BioBricks) with defined behaviors that can be assembled to construct complex biological devices [1]. The genetic parts sent to iGEM teams each year in the DNA distribution kit are derived from the Registry, and iGEM teams are expected to improve the Registry by further characterizing existing parts and by submitting new parts for inclusion. Here we provide evidence that current requirements on DNA sequences for part submission may unnecessarily impede this mission, and propose a new submission standard that would eliminate this problem while minimally impacting current quality control protocols.

As described in BioBricks Foundation RFC 10 and as currently used by the Registry, BioBricks constitute a combined assembly and submission standard. An assembly standard is a procedure for combining multiple biological parts into a device encoded by a single piece of DNA. A submission standard refers only to requirements on the DNA sequence of a part for it to be archived and redistributed by the Registry. BioBrick parts are typically submitted on the *Escherichia coli* plasmid pSB1C3, and they must be flanked by defined prefix and suffix sequences containing restriction enzyme sites for 3A assembly [2] (Figure 1A). Critically, these reserved (or “illegal”) restriction sites must not be present within the sequence of a BioBrick for it to be compatible with RFC 10 or similar assembly standards. The pSB1C3-derived plasmid can be transformed into *E. coli* cells to replicate the DNA encoding a part with high fidelity, and the quality and identity of each genetic part in the Registry can be verified by restriction analysis.

**Figure 1:**
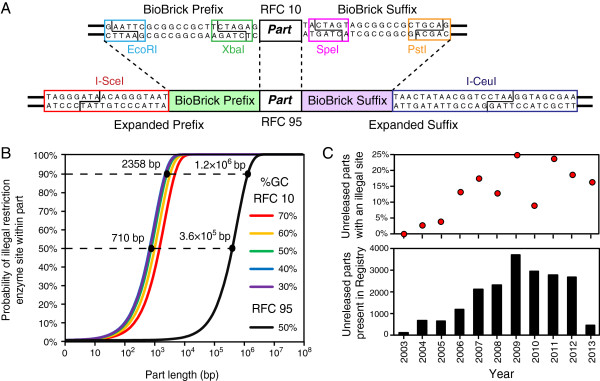
**Prevalence of illegal restriction sites in Biobrick parts. (A)** Restriction enzyme sites in the required BioBrick prefix and suffix sequences for RFC 10 are depicted above the expanded prefix and suffix sites with flanking homing endonuclease sites proposed in RFC 95. The four restriction enzyme sites EcoRI, XbaI, SpeI, and PstI contained within the BioBrick prefix and suffix must not be present within any part submitted in RFC 10 format. RFC 95 retains the Biobrick prefix and suffix and pSB1C3 plasmid backbone, but adds the homing endonuclease sites I-SceI and I-CeuI, which can be used for quality control. Recognition sites for the endonucleases are boxed and the cut sites are shown within the boxes. I-SceI and I-CeuI homing endonucleases tolerate some base substitutions in these sites, so the overall sequence degeneracy is roughly equivalent to that of a non-degenerate 10 to 12 bp restriction site [3]. **(B)** The probabilities of encountering at least one of the four RFC 10 BioBrick restriction enzymes sites (colored) or at least one of the RFC 95 homing endonuclease sites (black) in random DNA sequences as a function of sequence length are shown. The impact of variable GC content in the part sequence is depicted for the BioBrick restriction enzymes. Performing quality control for part length with homing endonucleases would nearly eliminate the probability of an illegal site being observed in a gene-sized DNA sequence. BBF RFC 95 contains the equations used to calculate the curves [4]. **(C)** The total number of DNA sequences in the Registry submitted in each year with a status of “Not Released” (lower) and the percentage of these parts that contain at least one RFC 10 illegal restriction site in their sequence (upper) is increasing with time, suggesting a significant and growing burden in adhering to this assembly standard. Data were collected for all parts submitted by July 29, 2013.

At the time BioBricks were introduced, restriction enzyme cloning was the dominant method for assembling multiple DNA sequences into a single construct, and *E. coli* was the host for nearly all synthetic biology devices. Since the inception of RFC 10, a great variety of new assembly methods have been developed [5, 6], including homology-based protocols using enzymes *in vitro* (Gibson Cloning, Seamless Cloning), *in vivo* assembly (via yeast recombination), and assembly using type II restriction enzymes (Golden Gate Assembly). Some of these methods can rapidly compose many parts together in a single reaction, unlike 3A assembly, which requires multiple rounds of restriction cleavage and ligation to concatenate parts. Many of these newer assembly methods also have no inherent requirement that specific base sequences, such as restriction sites, be present or absent in the DNA specifying a component in order for it to be assembled with other parts. When using such methods, there is no need for an assembly standard to be imposed on top of a submission standard. Researchers now employ synthetic biology approaches in many organisms, including plants and animals, where this greater flexibility in the sequences of vectors and genetic parts may be beneficial [7].

As genes and gene clusters with new activities are discovered and iGEM teams seek to add these parts to the Registry, greater incidences of illegal restriction enzyme site sequences are expected to be found within the DNA sequences of prospective parts. While the assembly standard’s requirement to remove any illegal restriction sites present in a part may seem a minor inconvenience, calculating the frequency at which restriction sites occur reveals that compliance with the BioBrick RFC 10 (or similar restriction enzyme-based standards) likely burdens most iGEM teams wishing to submit gene-sized or longer parts amplified from genomic sequences to the Registry (Figure 1B). The probability of a random DNA sequence containing at least one of the four BioBrick restriction sites increases rapidly with sequence length, such that a majority of parts derived from natural sequences >710 bp will contain a restriction site, and more than 90% of those >2360 bp will [4]. Furthermore, an analysis of parts marked “Not Released” in the Registry—often in this category because they do not adhere to the RFC 10 BioBrick standard and were therefore not accepted for archival and redistribution—shows that the fraction of parts designed by recent iGEM teams that contain an illegal site is >15% and appears to be increasing (Figure 1C).

In light of these developments, we argue that it would be beneficial to many iGEM teams and the greater biological engineering community to no longer require compatibility with assembly standards for a DNA part to be deposited in the Registry. To assess user sentiment, we surveyed the 2013 iGEM teams about their preferred assembly methods and experiences with submitting parts to the Registry (Figure 2). Though a majority of teams still primarily used restriction enzyme cloning, 43% most commonly used “one-pot” assembly methods rather than restriction enzyme cloning, showing that many teams are already adopting these newer methods. We found that 52% of teams surveyed have used site-directed mutagenesis to remove illegal restriction sites from parts, and 36% of teams have decided not to submit a part to the Registry due to the presence of illegal restriction sites. Thus, a sizable proportion of our respondents were expending time and effort performing site-directed mutagenesis of already functioning parts to comply with RFC 10—a substantial burden on the productivity of teams.

**Figure 2:**
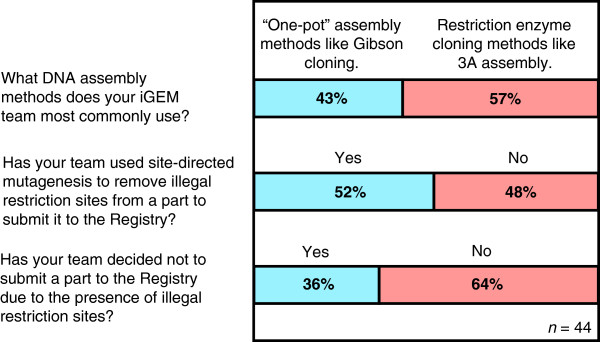
**Results of a survey sent to iGEM teams regarding illegal restriction sites.** Official contacts for all of the 2013 iGEM teams were emailed a link to an anonymous online survey. A total of 44 responses from iGEM team members and their mentors were collected and analyzed.

The Registry needs a submission standard that maintains a simple and rapid method for quality control of submitted parts. As described in RFC 95, this aim could be accomplished by using a less restrictive submission-only standard where homing endonuclease sites are included outside of the BioBrick prefix and suffix sequences [4]. Homing endonucleases recognize and cleave within long target sequences (~15-30 base pairs) in contrast to the short sequences (6-8 base pairs) recognized by most commonly used restriction enzymes. These longer recognition sequences are unlikely to occur in DNA sequences of <20 kilobases (Figure 1B), which is approximately the limit of what can be routinely cloned into plasmids in *E. coli*. By placing the homing endonuclease sites outside of the current BioBrick restriction enzyme sites, new parts submitted using this standard would remain backwards compatible with RFC 10 assembly in cases where no BioBrick restriction sites are found in the part.

While DNA synthesis methods are advancing rapidly [8, 9], making it more economical for iGEM teams to custom order a limited number of ready-to-use parts, the Registry continues to play an important role in democratizing synthetic biology by distributing a large number of parts at a much lower cost. For a genetic parts repository and registry to remain relevant as technology progresses, it should anticipate these changes and adapt its methods to complement them [10]. This may include adopting greater flexibility by decoupling DNA assembly and submission standards, as described here, as well as more rigorous and standardized expectations for how the operation of genetic parts must be characterized in order for them to be included in the Registry.

## References

[1] Canton B, Labno A, Endy D (2008). Refinement and standardization of synthetic biological parts and devices. Nat Biotechnol.

[2] Knight T: **Draft standard for BioBrick biological parts.** http://hdl.handle.net/1721.1/45138

[3] Gimble FS, Wang J (1996). Substrate recognition and induced DNA distortion by the PI-SceI endonuclease, an enzyme generated by protein splicing. J Mol Biol.

[4] Hammerling M, Gottel NR, Alnahas RN, Slater B, Huang Y, Okasheh Y, Howard M, Mortensen C, Monk J, Detelich M, Lannan RS, Pitaktong A, Weaver E, Das S, Barrick JE: **BBF RFC 95: Open Sequence Initiative: a part submission standard to complement modern DNA assembly techniques.** http://hdl.handle.net/1721.1/81334

[5] Ellis T, Adie T, Baldwin GS (2011). DNA assembly for synthetic biology: from parts to pathways and beyond. Integr Biol (Camb).

[6] Kahl LJ, Endy D (2013). A survey of enabling technologies in synthetic biology. J Biol Eng.

[7] Wang Y-H, Wei KY, Smolke CD (2013). Synthetic biology: advancing the design of diverse genetic systems. Annu Rev Chem Biomol Eng.

[8] Esvelt KM, Wang HH (2013). Genome-scale engineering for systems and synthetic biology. Mol Syst Biol.

[9] Ma S, Tang N, Tian J (2012). DNA synthesis, assembly and applications in synthetic biology. Curr Opin Chem Biol.

[10] Vilanova C, Porcar M (2014). iGEM 2.0–refoundations for engineering biology. Nat Biotechnol.

